# Education, Other Socioeconomic Characteristics Across the Life Course, and Fertility Among Finnish Men

**DOI:** 10.1007/s10680-017-9430-8

**Published:** 2017-07-27

**Authors:** Jessica Nisén, Pekka Martikainen, Mikko Myrskylä, Karri Silventoinen

**Affiliations:** 1 Population Research Unit, Department of Social Research, University of Helsinki, P.O. Box 18 (Unioninkatu 35), 00014 Helsinki, Finland; 2 Max Planck Institute for Demographic Research, Konrad-Zuse-Straße 1, 18057 Rostock, Germany; 3 Centre for Health Equity Studies (CHESS), Stockholm University, Stockholm, Sweden; 4 Karolinska Institutet, Stockholm, Sweden; 5 Department of Social Policy, London School of Economics, London, UK; 6 School of Medicine, Osaka University, Suita, Japan

**Keywords:** Education, Socioeconomic differences, Fertility, Male fertility, Childlessness, Parity progression, Within-family design

## Abstract

The level of education and other adult socioeconomic characteristics of men are known to associate with their fertility, but early-life socioeconomic characteristics may also be related. We studied how men’s adult and early-life socioeconomic characteristics are associated with their eventual fertility and whether the differences therein by educational level are explained or mediated by other socioeconomic characteristics. The data on men born in 1940–1950 (*N* = 37,082) were derived from the 1950 Finnish census, which is linked to later registers. Standard and sibling fixed-effects Poisson and logistic regression models were used. Education and other characteristics were positively associated with the number of children, largely stemming from a higher likelihood of a first birth among the more socioeconomically advantaged men. The educational gradient in the number of children was not explained by early socioeconomic or other characteristics shared by brothers, but occupational position and income in adulthood mediated approximately half of the association. Parity-specific differences existed: education and many other socioeconomic characteristics predicted the likelihood of a first birth more strongly than that of a second birth, and the mediating role of occupational position and income was also strongest for first births. Relatively small differences were found in the likelihood of a third birth. In men, education is positively associated with eventual fertility after controlling for early socioeconomic and other characteristics shared by brothers. Selective entry into fatherhood based on economic provider potential may contribute considerably to educational differentials in the number of children among men.

**Electronic supplementary material:**

The online version of this article (doi:10.1007/s10680-017-9430-8) contains supplementary material, which is available to authorized users.

## Introduction

Education may influence childbearing among men in various ways over the life course (Berrington and Pattaro 2014; Thomson et al. 2013). An economic mechanism is among the most commonly discussed mechanisms linking achieved educational level to fertility (Huinink 1995; Kravdal and Rindfuss 2008): higher educational attainment is expected to increase men’s fertility through higher income levels and better labour market positions (Becker 1993). Economic potential may also contribute to higher fertility among men through better chances in the marriage market (Becker 1993; Oppenheimer 1988; Oppenheimer et al. 1997). However, other mechanisms linking education to childbearing among men are also likely to be relevant, and the relative importance of different mechanisms may be sensitive to parity and the societal context. The number of children can be viewed to result from consecutive decisions in the life course (Kreyenfeld and Konietzka 2008; Thomson et al. 2013); fertility in this study comprises the eventual number of children and the chances of a first, second, and third birth.

In life-course research on family and fertility, researchers have called for more attention on potential early-life influences (Huinink and Feldhaus 2009; Huinink and Kohli 2014). Education plays a central role in the transmission of the socioeconomic standing of the previous generation to the next (Breen and Jonsson, 2005) and strongly determines other socioeconomic characteristics in adulthood (Elo 2009; Lynch and Kaplan 2000). Socioeconomic characteristics in early life have been previously linked with men’s fertility (Easterlin 1966; Thornton 1980). This study aims to extend the previous literature by carefully analysing to what extent educational differences in men’s fertility are explained by socioeconomic or other characteristics in early life, or mediated by such characteristics in adulthood. In addition to observed early characteristics, we control for unobserved characteristics shared by brothers. Further, we describe the relationships of socioeconomic characteristics in early life and adulthood with fertility.

Men’s fertility has traditionally attracted relatively little interest among demographers (Bledsoe et al. 2000; Forste 2002; Goldscheider and Kaufman 1996; Zhang 2011). Educational differentials in fertility are a widely studied topic, but previous literature has mostly focused on women (Balbo et al. 2013). This study aims at contributing to the understanding of the relations of education and other socioeconomic characteristics across the life course with fertility among men by utilizing longitudinal data on Finnish male cohorts born in the period 1940–1950. The context of childbearing for these birth cohorts implied an increasing popularity of the two-earner family model and government support for families, alongside continuously gendered views of the breadwinner and caregiver roles in society (Ellingsaeter and Leira 2006; Julkunen 1999).

## Conceptual Framework

Several mechanisms may link education to fertility in men, and the relative importance of different mechanisms may be sensitive to parity and the societal context. The most commonly discussed mechanism builds on the economic approach to fertility, assuming that individuals behave rationally and the demand for children increases at higher income levels (Becker 1993; Berk and Berk 1983; Pollak and Watkins 1993). This implies that higher acquired educational attainment leads to better chances of providing for a family with a larger number of children through accumulated human capital, higher income levels, and better labour market prospects. This income effect thus suggests a positive influence of education on fertility that operates through income and labour market position, regardless of parity. Yet at higher income levels, the opportunity costs of children can increase through forgone money and experience following reduced working hours (Becker 1993). In men, the positive effect of income is expected to dominate any negative effect of opportunity costs in contexts where men are considered main providers of the family income.

The strengthening of women’s labour market position is suggested to have been followed by men’s increasing involvement at home and a continuing change towards more symmetrical gender roles (Goldscheider et al. 2015; Hook 2006). Nordic countries, including Finland, have been forerunners in this respect, with such changes occurring earlier than elsewhere (Esping-Andersen 2009; Goldscheider et al. 2014). Despite increased expectations towards men’s involvement at home from the 1950s onwards, gender roles still remain somewhat asymmetrical, even in Nordic countries (Joshi 1998; Prince Cooke and Baxter 2010). Findings regarding time trends in socioeconomic differences of fertility do not necessarily indicate weakening expectations for men as economic providers (Hart 2015; Kravdal and Rindfuss 2008; Lappegård et al. 2011; Ravanera and Beaujot 2014; Winkler-Dworak and Toulemon 2007).

The traditional economic approach to family has been criticized for its underlying assumption of a gendered division of labour in the household (Esping-Andersen 2009; McDonald 2000; Oppenheimer 1997). Gender specialization in the strict sense of the term has not been the reality of industrialized countries in recent decades, but the concepts of income effect and opportunity cost are still relevant. The expected positive influence of men’s education on their fertility operating through an income effect could be counterbalanced particularly at higher parities if more highly educated parents decided to invest more in each of their children, thereby increasing the costs per child (Becker 1993; Becker and Lewis 1973). This phenomenon refers to the trade-off between the quantity and quality of children, with higher-income parents potentially preferring to have fewer children with higher quality regarding aspects such as children’s education and well-being.

Education may also relate to fertility among men through non-economic mechanisms, that is, mechanisms that do not operate via income and position in the labour market. For example, during educational enrolment, the incompatibility of student and parent roles is likely to constrain childbearing (Corijn and Klijzing 2001; Sigle-Rushton 2005). Yet given that men are less constrained by declines in fecundity with age (Billari et al. 2011; Schmidt et al. 2012), men have time to catch up on their childbearing after completing their studies, and the effect of enrolment is likely to be less decisive on their eventual fertility. Any negative effect of long-term enrolment could, however, be expected to be strongest for the first parity (Kravdal 2007).

Educational level may also reflect life values: post-materialist values more common among the more highly educated may be linked to weaker preferences for a large number of children and the seeking of fulfilment in life in alternative ways (Inglehart 1990). Further, the strength of the two-child norm may vary according to educational group, as, for example, stronger intentions towards having at least two children were witnessed among highly educated British men (Berrington and Pattaro 2014). Moreover, men at different educational levels may differ in their knowledge and practice of contraceptive behaviour, which may affect fertility, particularly at younger ages and lower parities (Nelson 2004).

Finally, given the scarce evidence (Baizán and Martín-García 2006; Martín-García 2009), it remains possible that the education–fertility association in men would be confounded by certain early influences that directly influence both education and fertility. Characteristics of the family of origin could confound the association if they influenced preferences and opportunities regarding education and family life (Axinn et al. 1994; Miller 1992, 1994; Thornton 1980). According to economic reasoning, material resources in the family of origin may discourage fertility because of the relatively high consumption aspirations adopted in childhood and adolescence (Easterlin 1966; Thornton 1980). These could be reflected in acquiring education at the cost of childbearing or in limiting the number of children to ensure the children’s sufficient quality. Additionally, life goals other than family building might be emphasized more in families of higher socioeconomic status (Rijken and Liefbroer 2009; Scott 2004), and the potential influence may extend to behavioural outcomes in the next generation.

The arguments introduced thus far have primarily concerned fertility decision-making among couples. Fertility is closely related to union formation and stability such that differentiating between cause and effect is ambiguous (Berrington and Pattaro 2014; Huinink 1995; Van Bavel et al. 2012). In the context of this study, marital unions could be primarily considered potential mediators of the education–fertility association. Economic approaches predict that men with better standing in the labour market are more successful in the marriage market (Becker 1993; Oppenheimer 1988; Oppenheimer et al. 1997). To some extent, this may relate to prospective childbearing: if economic resources are required for establishing an independent household and having children, and men are considered important family-income providers, then the more highly educated men with actual or prospective higher incomes and better labour market prospects may be viewed as more attractive partners and potential fathers by women.

Accordingly, a man’s higher education usually predicts both higher chances of marrying and marital stability (Lyngstad and Jalovaara 2010; Prince Cooke and Baxter 2010). In Finland, men educated to lower levels have been shown to be disadvantaged in both the formation (Finnäs 1995; Jalovaara 2012) and stability (Finnäs 1997; Jalovaara 2003) of marital unions. The experience of divorce is usually associated with lower fertility, but remarrying may increase the fertility of men (Van Bavel et al. 2012). Further, given the tendency of socioeconomic homogamy (Domanski and Przybysz 2007; Mäenpää 2015), the effect of female partner’s characteristics on educational differences in men’s fertility remains an important area of research (Begall 2013; Jalovaara and Miettinen 2013).

## Previous Findings

In Nordic countries, men educated to higher levels, at least in younger birth cohorts, less often remain childless and have higher numbers of children on average (Fieder and Huber 2007; Goodman and Koupil 2009; Kravdal and Rindfuss 2008; Lappegård et al. 2011; Nikander 1995; Nisén et al. 2014a; Rønsen and Skrede 2010). In other Western countries, the corresponding associations vary from positive to flat to negative (Barthold et al. 2012; Hopcroft 2015; Keizer et al. 2008; Kiernan 1989; Kneale and Joshi 2008; Nettle and Pollet 2008; Parr 2010; Ravanera and Beaujot 2014; Skirbekk 2008; Thomson et al. 2013; Toulemon and Lapierre-Adamcyk 2000; Toulemon et al. 2008; Tragaki and Bagavos 2014; Weeden et al. 2006). A recent comparative study reported childlessness at the ages of 40–44 to be more common among men educated to lower levels in 13 out of 19 European countries (Miettinen et al. 2015).

Previous studies show that men enrolled in education have low chances of experiencing a childbirth (e.g. Dribe and Stanfors 2009; Kravdal 2007; Thalberg 2013). A higher level of education in turn, as estimated often after controlling for educational enrolment and a few other socioeconomic characteristics in adulthood, has been found to predict both higher (Hart 2015; Lappegård and Rønsen 2013; Winkler-Dworak and Toulemon 2007) and lower (Guzzo and Furstenberg 2007; Liefbroer and Corijn 1999; Martín-García 2009; Özcan et al. 2010) entry rates into fatherhood, and some studies document no differences (Dribe and Stanfors 2009; Huinink 1995; Özcan et al. 2010) or a U-shaped pattern (Tölke and Diewald 2003).^1^ Apart from education, higher income and often a stronger attachment to the labour market tend to associate with higher entry rates into fatherhood (Hart 2015; Huinink 1995; Kravdal 2002; Kreyenfeld and Andersson 2014; Lappegård and Rønsen 2013; Liefbroer and Corijn 1999; Özcan et al. 2010; Pailhé and Solaz 2012; Schmitt 2012; Tölke and Diewald 2003; Winkler-Dworak and Toulemon 2007).

Studies on higher-order birth rates among men suggest mainly positive associations with educational level in Nordic countries (Duvander and Andersson 2006; Duvander et al. 2010; Kravdal 2007; Kravdal and Rindfuss 2008; Lappegård and Rønsen 2013; Thomson et al. 2013), but not necessarily elsewhere (Bronte-Tinkew et al. 2009; Guzzo and Furstenberg 2007; Oláh 2003). For example, in Norway, educational level stimulated higher-order births, after controlling for enrolment and some background characteristics (Kravdal 2007), or for enrolment, income, and parental education (Lappegård and Rønsen 2013). Moreover, a well-educated male partner tends to increase second birth rates among women (Bartus et al. 2013; Gerster et al. 2007; Kreyenfeld 2002). With respect to male income and labour market attachment, there is evidence of a positive effect on second but not necessarily third births (Andersson and Scott, 2007; Kravdal 2002; Kreyenfeld and Andersson 2014; Lappegård and Rønsen 2013; Pailhé and Solaz 2012).^2^

Studies analysing educational differences in fertility often control for some family-of-origin characteristics, such as parental education, family type, and level of urbanization, with little attention paid to them (e.g. Huinink 1995; Kravdal and Rindfuss 2008; Liefbroer and Corijn 1999; Winkler-Dworak and Toulemon 2007). The literature on early-life predictors of childbearing associates a higher socioeconomic position of a parent with later entry into parenthood (e.g. Dahlberg 2015; Dribe and Stanfors 2009; Hynes et al. 2008; Thornton 1980), but the respective findings regarding the eventual number of children of men vary (Goodman and Koupil 2009; Murphy and Wang 2001; Parr 2010; Rijken and Liefbroer 2009). Further, associations with men’s fertility have been found for other than socioeconomic characteristics of the family of origin, such as number of siblings and religiosity (Kolk 2014; Murphy and Wang 2001; Rijken and Liefbroer 2009).

Some previous studies indicate differences between parities with respect to education (Guzzo and Furstenberg 2007; Kravdal and Rindfuss 2008); in Norway, a stronger effect on first than higher-order births appeared (Lappegård and Rønsen 2013). Also the association of other socioeconomic characteristics with fertility in men may depend on the parity in question (Kravdal 2002; Kreyenfeld and Andersson 2014; Lappegård and Rønsen 2013; Pailhé and Solaz 2012). Little is known on potential parity differences regarding early-life socioeconomic characteristics.

## Aims and Context of the Study

The role of education and other socioeconomic characteristics in men’s fertility has been addressed in the previous literature, but the mechanisms behind educational differences are still not entirely clear. Economic mechanisms related to income and position in the labour market are often discussed, but alternative mechanisms remain plausible with potentially varying importance depending on parity and the societal context. We conceptualize occupational position as a more proximate indicator of earning potential and attachment to the labour market than education, whereas income measures actual earnings and is a strong indicator of economic resources overall (Elo 2009; Lynch and Kaplan 2000). To gain both overall and parity-specific understanding, fertility in this study comprises the eventual number of children and chances of a first, second, and third birth. The research questions are:

(i) How do the level of education, occupational position, income, and early-life socioeconomic characteristics associate with fertility in men?
(ii) Are educational differences in men’s fertility explained by early-life socioeconomic or other characteristics shared by brothers?
(iii) Are educational differences in men’s fertility mediated by occupational position and income in adulthood?

We expect to find positive associations between men’s socioeconomic characteristics in adulthood and fertility, but do not hypothesize about the respective associations with early socioeconomic characteristics (i). Regarding educational differences, we expect any explanatory role of early characteristics to be weaker (ii) than any mediating role of adult characteristics (iii). Parity differences, if any, are expected to show as weaker associations of socioeconomic characteristics with higher-order births. We build on the current literature by studying the role of education in men’s childbearing more thoroughly in relation to other socioeconomic characteristics across the life course. The study is based on Finnish population-based register data on birth cohorts 1940–1950 with detailed non-retrospective measurements of early-life characteristics. The data uniquely allow follow-up of the early-life stages up until late reproductive ages, a rich non-retrospective measurement of early-life socioeconomic characteristics, and sibling comparison.

The context of this study implied low living standards in the mid-twentieth century, but rising levels thereafter (Jäntti et al. 2006). When the men studied here were born, Finland was a poor country that was at war or recovering from it. Later, in the second half of the century, the overall living standards rose rapidly due to economic growth and structural change. Concurrently, the publicly provided welfare support for families increased as part of the welfare state expansion (Rønsen 2004), and women and men born between 1940 and 1950 witnessed this increasingly during their prime childbearing years. The Finnish society is often described as having relatively low social inequality in terms of income (Jäntti et al. 2006), and the Finnish educational system is considered flexible and socially inclusive (Orr et al. 2011).

Finland is characterized by a relatively strong dual-earner family model (e.g. separate taxation of the husband and wife since 1976) (Aarnio and Eriksson 1987). In 1970, 39% of married women aged 24–54 were housewives, whereas in 1980 correspondingly only 10% (Julkunen 1999). The labour force participation of women with preschool children was already high in the 1950s and 1960s, and the share of employed mothers working part-time low even in Nordic comparisons (Rønsen and Sundstrom 2002). The heavier burden of breadwinning still continued to fall on men’s shoulders, as exemplified in 1982 when men’s earnings comprised over 60% of total household earnings among married dual-earner couples (Aarnio and Eriksson 1987). In the studied birth cohorts, men were still educated to higher levels than women (Havén 1999).

Despite the high share of dual-earner families in Finland in the 1970s, the role of men in housework and childrearing remained limited. In the 1970s, a more equal division of labour between mothers and fathers was facilitated by legislation, and Finnish fathers have been eligible to take parental leave since 1978 (Ellingsaeter and Leira 2006; Haataja 2004). Yet by then over half of the male cohort under study had already become fathers (Nisén et al. 2014a). The initial 2-week leave that fathers were entitled to was later extended (Haataja 2004). Fathers’ role in childcare still continued to be weak compared to women: in 1990 fathers took only 2–3% of all parental leave days in Finland (Ellingsaeter and Leira 2006; Haataja 2004).

## Methods

### Data and Variables

The data were obtained from a 10% sample of households drawn from the 1950 Finnish census (permission TK-53-704-10) (Statistics Finland 1997). Information on members of the sampled households was subsequently linked to sociodemographic information from quinquennial censuses in 1970–1995 and to the Finnish Population Register for fertility histories. We restricted the data to the 1940–1950 birth cohorts. The original sample consisted of 411,628, of whom 91,452 were born between 1940 and 1950 and lived in a one- or two-parent family at the time of the census in 1950 (46,782 men). Respondents were excluded with missing information on childhood variables or absent from the census at the ages of 30–34 (*n* = 7417), lost to follow-up at the ages of 45–49 (*n* = 2281), or with an unrealistic age at first birth (*n* = 2). Loss to follow-up was attributable to emigration, mainly to Sweden in the late 1960s and the early 1970s, and to a lesser extent to mortality between 1950 and 1990/1995. The final study sample included 37,082 men. Brothers were identified based on information on place of residence, household, and family collected in 1950. The analysed men came from 27,305 families, of which at least two male siblings were identified in 7671 families. This identification procedure did not distinguish between biological and non-biological siblings. Siblings who had died, moved out of the parental home, or were not yet born at the time of the census were not covered.^3^

Monthly information on live-born biological children was linked to the data from birth records from 1970 to 2009. Children born before 1970 were included, except in cases where they did not live with their fathers around the year 1970 when personal identification codes were introduced. The study participants were 59–69 years old at the end of the follow-up in 2009. In these data, the fertility of men (*M* 1.81, SD 1.45) was close to that of women in a corresponding sample (*M* 1.85, SD 1.38) (Nisén et al. 2014b). Thus, we expect bias from unknown paternity to be small. In addition to the total number of children, the likelihood of a first, second, and third birth was analysed.

The socioeconomic characteristics in adulthood comprised level of education, occupational position, and income. These variables were measured at one point in time at the age of 30–34 based on census information from the years 1970, 1975, or 1980. The main explanatory variable, the level of education, was categorized into four classes: basic, lower secondary, upper secondary, and tertiary (Table 1). The basic level refers to a maximum of 9 years of mandatory education. The lower-secondary level refers to brief vocational training (<3 years) undertaken in addition to basic education. Upper-secondary education refers to either academic education (matriculation) or vocational training (≥3 years) undertaken in addition to basic education. The tertiary level refers either to a university degree or to vocational training at the highest level (≥4 years after general education). Occupational position was classified as manual worker, lower white collar, upper white collar, farmer/self-employed (64% farmers), or other/unknown. For income, the values from different years of taxable income reported in the census were first converted into income in 2012 (Statistics Finland 2013) and then divided into quintiles. In the sample, 3% had no income.

**Table 1:** Descriptive statistics of the study population: Finnish men, *N* = 37,082

	Parity							
	0 (SE)	1 (SE)	2 (SE)	3 (SE)	4 (SE)	5 + (SE)	Total	*N*
*Parity distribution by level of education (%)*								
Level of education								
Basic	23.8 (0.003)	18.7 (0.003)	33.6 (0.004)	15.9 (0.003)	5.4 (0.002)	2.6 (0.001)	100.0	16,561
Lower secondary	18.0 (0.004)	19.7 (0.004)	38.3 (0.005)	16.7 (0.004)	5.2 (0.002)	2.2 (0.002)	100.0	10,275
Upper secondary	14.3 (0.005)	17.8 (0.005)	43.2 (0.007)	17.8 (0.005)	5.0 (0.003)	2.0 (0.002)	100.0	5073
Tertiary	12.1 (0.005)	14.8 (0.005)	42.1 (0.007)	22.4 (0.006)	6.1 (0.003)	2.5 (0.002)	100.0	5173
Total	19.3 (0.002)	18.3 (0.002)	37.4 (0.003)	17.3 (0.002)	5.4 (0.001)	2.4 (0.001)	100.0	37,082
*N*	7139	6791	13,865	6407	1997	883	37,082	

	%	*M*	SD		%	*M*	SD		%	*M*	SD
*Distribution of explanatory variables (%) and number of children accordingly (M, SD)*											
Living area in childhood				Parental level of education				Level of education			
Helsinki region	8.0	1.76	1.23	Less than primary	13.5	1.75	1.70	Basic	44.7	1.71	1.50
Rest of Uusimaa	5.7	1.75	1.30	Primary school	75.6	1.81	1.42	Lower secondary	27.7	1.80	1.31
Western Finland	39.6	1.83	1.34	More than primary	10.9	1.90	1.30	Upper secondary	13.7	1.90	1.30
Eastern Finland	42.3	1.81	1.58	Parental occupational position				Tertiary	14.0	2.06	1.35
Northern Finland	4.4	1.86	1.66	Worker	42.5	1.76	1.40	Occupational position			
Family type in childhood				Professional/administrative	16.0	1.84	1.31	Manual worker	47.0	1.73	1.39
Two parents and children	93.0	1.82	1.46	Farmer, <10 hect.	24.7	1.82	1.62	Lower white collar	18.5	1.91	1.23
Mother and children	6.2	1.72	1.33	Farmer, ≥10 hect.	7.8	1.94	1.50	Upper white collar	16.2	2.07	1.30
Father and children	0.8	1.61	1.34	Self-employed/other/unknown	9.0	1.87	1.39	Farmer/self-employed	10.3	2.04	1.60
Sibship size								Other/unknown	8.1	1.26	1.47
0	15.3	1.72	1.25	Parental home ownership				Income			
1–2	48.1	1.81	1.37	Owner	59.3	1.82	1.48	1st quintile	20.0	1.47	1.54
3–	36.6	1.85	1.63	Renter	34.7	1.79	1.34	2nd quintile	20.0	1.82	1.29
Year of birth				Other, unknown	6.1	1.80	1.75	3rd quintile	20.0	1.87	1.38
1940	6.3	1.84	1.28	Crowding in childhood				4th quintile	20.0	1.87	1.31
1941	8.7	1.86	1.33	<2	32.9	1.84	1.29	5th quintile	20.0	2.03	1.29
1942	6.1	1.81	1.34	2 < 3	32.5	1.81	1.48	Marital history			
1943	7.4	1.83	1.36	≥3	34.6	1.78	1.57	Never married	17.5	0.53	1.04
1944	7.9	1.83	1.33	Standard of living childhood				Intact married	51.9	2.09	1.38
1945	9.6	1.82	1.30	Poor	28.2	1.78	1.60	Divorced/widowed	19.7	1.92	1.15
1946	11.1	1.79	1.30	Modest	46.4	1.81	1.45	Remarried	10.9	2.36	1.34
1947	11.2	1.78	1.35	Good	25.4	1.85	1.27				
1948	11.0	1.78	1.36								
1949	10.5	1.82	1.44								
1950	10.3	1.82	1.44								

M: mean, SD: standard deviation, SE: standard error

The early-life socioeconomic characteristics included parental education, parental occupational position, and measures of overall living conditions. These were measured at one point in time in the 1950 census when the men were between the ages of zero and 10. The parental level of education measures the highest qualification achieved by either parent (74% of parents possessed the same level), categorized as less than primary school, primary school, and more than primary school. The parental occupational position was categorized as manual worker, professional or administrative, farmer with <10 hectares of land, farmer with ≥10 hectares of land, and self-employed/other. The variables measuring overall living conditions included parental home ownership (owner, renter, other, or unknown), crowding (number of persons per heated room: <2, 2 < 3, ≥3), and standard of living (poor, modest, good) in childhood. In this approximate measure, the category *poor* referred to households with no modern facilities such as electric lights, *modest* to households with one item, and *good* to those with at least two items.

The control variables (year of birth, sibship size, and family type and living area in childhood) were also measured at one point in time in the 1950 census when the men were between the ages of zero and 10. Family type was categorized as two parents with children, mother and children, and father and children; sibship size was divided into three categories (0, 1–2, 3–).^4^ The living area covered five geographical areas: the Helsinki (capital) region, the rest of Uusimaa (the area surrounding the capital region in the south part of Finland), western Finland, and eastern and northern Finland, which were both mainly agricultural areas in 1950.

Marital history was categorized as never married, intact married (first marriage not dissolved due to divorce or the partner’s death), divorced/widowed (87% divorced), or remarried. This classification was based on longitudinal information on the formation and dissolution of marital unions until 2009. Data on marriages formed and dissolved before 1970 were unavailable. Longitudinal information on cohabitation was not available, but it was still very uncommon in the birth cohort under study, becoming more common in Finland from the 1970s (Finnäs 1993).

### Statistical Analyses

Standard Poisson regression was used to assess the associations of education and other socioeconomic characteristics with the number of children. Standard logistic regression was used to study the associations of these explanatory characteristics with the likelihood of a first, second, and third birth. The full sample of men (*N* = 37,082) was used in the analysis of the number of children and the likelihood of a first birth. The likelihood of a second birth was analysed among fathers (*n* = 29,943), and the likelihood of a third birth among fathers with at least two children (*n* = 23,152).

We estimated nested standard regression models of all four fertility outcomes using the following strategy (Tables 2, 3; for full models of Table 3 see Supplementary material 1, 2, 3). In Model 0, the year-of-birth-adjusted associations of socioeconomic characteristics with fertility are estimated (a separate model for each socioeconomic characteristic). In Model 1, the education–fertility association was additionally adjusted for other control variables than year of birth: living area and family type in childhood, and sibship size. In Model 2, the association was adjusted additionally for socioeconomic characteristics in early life: parental education, occupational position and home ownership, and crowding and standard of living in childhood. Models 3 and 4 add occupational position and income in adulthood, respectively. Finally, marital history was added to Model 5.

**Table 2:** Incidence rate ratios (IRR) of the number of children among Finnish men, *N* = 37,082

Model	0	1	2	3	4	5
	IRR (SE)	IRR (SE)	IRR (SE)	IRR (SE)	IRR (SE)	IRR (SE)
Level of education						
Basic (ref.)	1	1	1	1	1	1
Lower secondary	1.05* (0.010)	1.06* (0.010)	1.06* (0.010)	1.05* (0.010)	1.03* (0.010)	0.99 (0.009)
Upper secondary	1.11* (0.012)	1.12* (0.011)	1.12* (0.011)	1.07* (0.013)	1.04* (0.013)	1.01 (0.012)
Tertiary	1.20* (0.013)	1.22* (0.011)	1.22* (0.012)	1.13* (0.017)	1.08* (0.017)	1.05* (0.016)
Living area in childhood						
Helsinki region	0.96* (0.014)	0.95* (0.014)	0.96* (0.015)	0.96* (0.015)	0.95* (0.015)	0.96* (0.014)
Rest of Uusimaa	0.96* (0.017)	0.97 (0.017)	0.97* (0.017)	0.96* (0.017)	0.96* (0.017)	0.97 (0.015)
Western Finland (ref.)	1	1	1	1	1	1
Eastern Finland	0.99 (0.008)	0.99 (0.009)	1.00 (0.010)	1.00 (0.010)	1.01 (0.010)	1.02* (0.009)
Northern Finland	1.02 (0.023)	1.01 (0.023)	1.03 (0.024)	1.04 (0.023)	1.04 (0.023)	1.07* (0.021)
Family type in childhood						
Two parents and children (ref.)	1	1	1	1	1	1
Mother and children	0.94* (0.017)	0.96* (0.017)	0.96* (0.017)	0.97 (0.017)	0.98 (0.016)	1.01 (0.014)
Father and children	0.88* (0.050)	0.90* (0.050)	0.91* (0.050)	0.92 (0.050)	0.92 (0.050)	0.93 (0.043)
Sibship size						
0 (ref.)	1	1	1	1	1	1
1–2	1.05* (0.011)	1.05* (0.011)	1.05* (0.012)	1.05* (0.011)	1.04* (0.011)	1.04* (0.010)
3	1.07* (0.013)	1.09* (0.013)	1.08* (0.014)	1.09* (0.014)	1.08* (0.014)	1.08* (0.012)
Parental level of education						
Less than primary (ref.)	1		1	1	1	1
Primary school	1.03* (0.015)		1.02 (0.015)	1.01 (0.015)	1.00 (0.015)	0.99 (0.014)
More than primary	1.09* (0.018)		1.04 (0.020)	1.03 (0.020)	1.03 (0.020)	1.01 (0.018)
Parental occupational position						
Worker (ref.)	1		1	1	1	1
Professional/administrative	1.04* (0.011)		0.97* (0.013)	0.96* (0.013)	0.97* (0.013)	0.97* (0.012)
Farmer, <10 hect.	1.03* (0.012)		1.03* (0.013)	1.01 (0.013)	1.02 (0.013)	1.03* (0.012)
Farmer, ≥10 hect.	1.10* (0.016)		1.07* (0.017)	1.03 (0.017)	1.04* (0.017)	1.05* (0.015)
Self-employed/other/unknown	1.06* (0.015)		1.04* (0.015)	1.03* (0.015)	1.04* (0.015)	1.03* (0.013)
Parental home ownership						
Owner (ref.)	1		1	1	1	1
Renter	0.98 (0.009)		0.99 (0.011)	1.00 (0.011)	1.00 (0.011)	1.00 (0.010)
Other/unknown	0.99 (0.021)		1.00 (0.021)	1.00 (0.021)	1.00 (0.021)	1.00 (0.018)
Crowding in childhood						
<2 (ref.)	1		1	1	1	1
2 < 3	0.98 (0.010)		1.00 (0.011)	1.00 (0.011)	1.00 (0.011)	1.01 (0.010)
≥3	0.97* (0.010)		1.00 (0.013)	1.01 (0.013)	1.01 (0.013)	1.01 (0.011)
Standard of living in childhood						
Poor (ref.)	1		1	1	1	1
Modest	1.02 (0.011)		1.02 (0.013)	1.01 (0.013)	1.00 (0.013)	0.99 (0.011)
Good	1.04* (0.011)		1.02 (0.015)	1.01 (0.015)	1.00 (0.015)	0.98 (0.013)
Occupational position						
Manual worker (ref.)	1			1	1	1
Lower white collar	1.11* (0.011)			1.09* (0.011)	1.07* (0.011)	1.02 (0.010)
Upper white collar	1.20* (0.012)			1.13* (0.016)	1.09* (0.015)	1.04* (0.015)
Farmer/self-employed	1.18* (0.016)			1.17* (0.014)	1.24* (0.014)	1.20* (0.013)
Other/unknown	0.73* (0.016)			0.73* (0.022)	0.82* (0.023)	0.92* (0.019)
Income						
1st quintile	0.77* (0.015)				0.80* (0.016)	0.93* (0.014)
2nd quintile	0.94* (0.012)				0.97* (0.012)	0.98* (0.011)
3rd quintile (ref.)	1				1	1
4th quintile	1.03* (0.012)				1.03* (0.012)	1.00 (0.011)
5th quintile	1.13* (0.012)				1.09* (0.012)	1.03* (0.011)
Marital history						
Never married	0.25* (0.025)					0.26* (0.026)
Intact married (ref.)	1					1
Divorced/widowed	0.92* (0.008)					0.94* (0.008)
Remarried	1.13* (0.010)					1.16* (0.010)

Model 0: explanatory variable + year of birth. Calculated separately for each explanatory variable

Model 1: level of education + control variables

Model 2: Model 1 + socioeconomic characteristics in early life

Model 3: Model 2 + occupational position

Model 4: Model 3 + income

Model 5: Model 4 + marital history

Method: Poisson regression analysis. In all models, year of birth is included as a continuous variable, but the coefficient is not shown

SE: standard error

An asterisk indicates when the 95% confidence interval does not include 1

**Table 3:** Odds ratios (OR) of the likelihood of a first, second, and third birth among Finnish men

Model	0	1	2	3	4	5
	OR (SE)	OR (SE)	OR (SE)	OR (SE)	OR (SE)	OR (SE)
*Odds ratios (OR) of the likelihood of a first birth among Finnish men, N* = *37,082*						
Level of education						
Basic (ref.)	1	1	1	1	1	1
Lower secondary	1.45* (0.021)	1.44* (0.021)	1.42* (0.021)	1.35* (0.021)	1.25* (0.033)	1.07 (0.042)
Upper secondary	1.90* (0.027)	1.87* (0.027)	1.84* (0.028)	1.34* (0.030)	1.19* (0.052)	1.00 (0.065)
Tertiary	2.28* (0.027)	2.24* (0.028)	2.22* (0.031)	1.41* (0.038)	1.13 (0.070)	0.90 (0.084)
*Odds ratios (OR) of the likelihood of a second birth among Finnish fathers, n* = *29,943*						
Level of education						
Basic (ref.)	1	1	1	1	1	1
Lower secondary	1.04 (0.033)	1.04 (0.034)	1.04 (0.034)	1.05 (0.034)	1.04 (0.034)	1.02 (0.035)
Upper secondary	1.25* (0.043)	1.29* (0.044)	1.27* (0.045)	1.21* (0.050)	1.18* (0.050)	1.15* (0.051)
Tertiary	1.61* (0.047)	1.68* (0.048)	1.63* (0.051)	1.45* (0.065)	1.37* (0.065)	1.33* (0.065)
*Odds ratios (OR) of the likelihood of a third birth among Finnish fathers of at least two children, n* = *23,152*						
Level of education						
Basic (ref.)	1	1	1	1	1	1
Lower secondary	0.88* (0.034)	0.89* (0.034)	0.89* (0.034)	0.92* (0.034)	0.93* (0.034)	0.93* (0.035)
Upper secondary	0.80* (0.041)	0.82* (0.042)	0.83* (0.043)	0.91* (0.049)	0.92 (0.049)	0.92 (0.050)
Tertiary	1.03 (0.040)	1.07 (0.040)	1.07 (0.044)	1.14* (0.058)	1.17* (0.059)	1.20* (0.060)

Model 0: explanatory variable + year of birth

Model 1: level of education + control variables

Model 2: Model 1 + socioeconomic characteristics in early life

Model 3: Model 2 + occupational position

Model 4: Model 3 + income

Model 5: Model 4 + marital history

Method: logistic regression analysis. In all models, year of birth is included as a continuous variable, but the coefficient is not shown

SE: standard error

An asterisk indicates when the 95% confidence interval does not include 1

An alternative to the standard Poisson model specification for analysing the number of children is the negative binomial model, which would have been preferable had there been overdispersion. However, evidence of overdispersion was not found: in the full (corresponding to Model 5 in Table 2) negative binomial model, the parameter indicating overdispersion did not differ from zero (*α* = 2.7 × 10^−8^), and the model gave virtually the same results as the Poisson model. We also considered the zero-inflated Poisson model as an alternative to the standard Poisson model, but preferred the latter based on the very few differences in predicted numbers of children between the models and the greater simplicity of the standard Poisson model.

Conditional sibling fixed-effects (FE) versions of the Poisson and logistic regression models were used to study whether the education–fertility association was confounded by unobserved characteristics shared by brothers (Table 4). This approach uses the family indicator included in the dataset to capture unobserved family characteristics and estimates the model parameters of education from variation between brothers. Thus, the FE models account for the family environment and genetic characteristics to the extent that these are shared by brothers, but at the cost of restricting the sample because those without a brother are excluded. Further, brother sets where all brothers had zero children are excluded in the Poisson FE model, and those where all brothers had the same outcome are excluded in the logistic FE models. The analysed brother sets include brothers born between 1940 and 1950, and alive and living in the same household in the 1950 census. These FE models were constructed by conditional maximum likelihood estimation (Allison 2009). Table 4 also shows estimates from Models 0–2 run in the samples used in the FE analysis to enhance comparability across models.

**Table 4:** Estimates from standard and sibling fixed-effects (FE) regression models in the subsamples of Finnish men used in the FE estimation

Model	0	1	2	FE
	IRR (SE)	IRR (SE)	IRR (SE)	IRR (SE)
*Incidence rate ratios (IRR) of the number of children, n* = *16,691* ^*a,c*^				
Level of education				
Basic (ref.)	1	1	1	1
Lower secondary	1.03* (0.014)	1.03* (0.014)	1.03* (0.014)	1.07* (0.019)
Upper secondary	1.10* (0.017)	1.11* (0.017)	1.12* (0.018)	1.11* (0.026)
Tertiary	1.17* (0.017)	1.18* (0.018)	1.19* (0.019)	1.19* (0.028)

Model	0	1	2	3
	OR (SE)	OR (SE)	OR (SE)	OR (SE)
*Odds ratios (OR) of the likelihood of a first birth, n* = *5875* ^*b,c*^				
Level of education				
Basic (ref.)	1	1	1	1
Lower secondary	1.36* (0.059)	1.38* (0.060)	1.40* (0.060)	1.54* (0.070)
Upper secondary	1.51* (0.085)	1.57* (0.086)	1.66* (0.091)	1.97* (0.108)
Tertiary	1.65* (0.077)	1.75* (0.078)	1.97* (0.091)	2.44* (0.121)
*Odds ratios (OR) of the likelihood of a second birth, n* = *4417* ^*b,c*^				
Level of education				
Basic (ref.)	1	1	1	1
Lower secondary	1.12 (0.069)	1.14 (0.070)	1.16* (0.070)	1.17 (0.083)
Upper secondary	1.24* (0.091)	1.29* (0.093)	1.35* (0.097)	1.41* (0.110)
Tertiary	1.36* (0.084)	1.43* (0.086)	1.55* (0.098)	1.65* (0.132)
*Odds ratios (OR) of the likelihood of a third birth, n* = *4141* ^*b,c*^				
Level of education				
Basic (ref.)	1	1	1	1
Lower secondary	0.93 (0.071)	0.93 (0.071)	0.93 (0.073)	0.90 (0.085)
Upper secondary	0.91 (0.087)	0.91 (0.088)	0.91 (0.093)	0.87 (0.113)
Tertiary	1.03 (0.075)	1.03 (0.079)	1.04 (0.093)	1.03 (0.122)

Model 0: level of education + year of birth

Model 1: level of education + control variables

Model 2: Model 1 + socioeconomic characteristics in early life

FE Model: level of education + year of birth + unobserved fixed family characteristics

SE: standard error

An asterisk indicates when the 95% confidence interval does not include 1

^a^Method: Poisson regression analysis

^b^Method: logistic regression analysis

^c^Estimates of other explanatory variables than education are not shown. In Models 0–2, explanatory variables are included as in Tables 2 and 3: all variables except year of birth are included as categorical variables

Throughout the analysis, we accounted for the clustering of brothers within families in the calculation of variance-based measures. We used the bootstrap procedure with cluster resampling in calculating the standard errors (SE) and 95% confidence intervals (with significant findings indicated by asterisks) in Tables 2, 3, 4, with 1000 replications and sibling sets as the clusters. The estimates of the Poisson regression models are reported as incidence rate ratios (IRR) and those of the logistic regression models as odds ratios (OR). The Stata statistical package, version 14 (StataCorp 2015), was used for the statistical analysis.

## Results

The men had on average 1.81 (SD 1.45) children in their lifetime, with 81% having at least one child. The parity distribution by level of education and other descriptive characteristics of the study population are shown in Table 1. Two was the most common number of children across educational groups (37%), with higher shares among men educated to upper-secondary and tertiary levels (42–43%). A large share of the men (45%) were educated to the basic level in this cohort, and only 14% had acquired tertiary education. Most men came from families with parental education at most at the primary level (76%) and from households with poor or modest living standards (75%). Manual worker was clearly the largest occupational group (47%).

Table 2 presents the results on the number of children. A clear positive gradient was apparent, with men educated at the tertiary level having 20% and at the upper-secondary level 11% more children than those educated at the basic level (Model 0). Other socioeconomic characteristics in adulthood also predicted fertility; men of higher occupational positions, farmers or self-employed, and especially those with higher incomes had a larger number of children on average. Additionally, socioeconomic characteristics in early life predicted fertility, higher parental education and occupational position, and less crowded and better-equipped childhood housing associated with higher fertility. The associations with the early characteristics were, however, modest and weaker than the associations with the adult characteristics.

Including additional control variables (living area and family type in childhood and sibship size) (Model 1) did not change the year-of-birth-adjusted association between education and fertility. Likewise, adjusting for early socioeconomic characteristics (Model 2) had no effect on these estimates. In turn, adjustments to occupational position (Model 3) and income (Model 4) clearly attenuated the differences by education by 41–68%.^5^ A weak positive association remained after all adjustments, with the tertiary-educated men having 8% more children than those with basic education. Accounting additionally for marital history (Model 5) further reduced the estimates of education, with now only tertiary-educated men having 5% more children compared to basic-educated men.

Results from the parity-specific analysis are shown in Table 3. Higher education, higher occupational position, and especially higher income all clearly predicted a higher likelihood of a first birth in the model adjusted for year of birth only (Model 0). Favourable socioeconomic characteristics in early life also predicted higher chances of a first birth, even if less strongly than characteristics in adulthood. The educational differences in the likelihood of a first birth remained after adjusting for other control variables (Model 1) and early-life socioeconomic characteristics (Model 2). The adjustment for occupational position (Model 3) and income (Model 4) again strongly, by 40–90%, attenuated the differences by education. When marital-history differences had been adjusted for (Model 5), no significant differences by education remained.^6^

Education and other socioeconomic characteristics in adulthood also predicted higher chances of a second birth, even if less strongly in comparison with first births, in the year-of-birth-adjusted models (Model 0). Some of the favourable characteristics that predicted higher chances of a first birth had a similar effect on second births. Adjustment for other control variables (Model 1) and socioeconomic characteristics in early life (Model 2) had a negligible effect on the estimates of education on second-birth likelihood, but adjustment for occupational position and income in adulthood (Models 3–4) attenuated the differences between the upper-secondary and tertiary-educated groups and the basic-educated group by 33–41%. The adjustment for marital history had a marginal effect, and the highly educated men were still more likely to experience a second birth (Model 5).

Differences by education in the likelihood of a third birth were small: in the year-of-birth-adjusted model the fertility of the lowest and highest educated was at the same level and that of men in the middle categories slightly lower (Model 0). Occupational position had a weak positive association, whereas men in the lowest income quintile were more likely to experience a third birth than two-child fathers with higher incomes. Most of those favourable early-life socioeconomic characteristics that predicted higher chances of a first, and to some extent a second birth, had a null or weak negative association with third-birth likelihood. The adjustment for controls and early-life socioeconomic characteristics had no effect on the education estimates (Models 1–2), and the adjustment for occupational position and income even slightly increased the estimate of the tertiary educated (Models 3–4). The adjustment for marital history had a marginal effect, and after the adjustment, a weak nonlinear association of education with the third-birth likelihood remained (Model 5).

Additionally, we note that while staying unmarried was associated with low fertility, remarried men showed the highest numbers of children and the highest chances of a first and especially a third birth (Table 2; Supplementary material 1, 2, 3). Divorced or widowed men had lower numbers of children and were less likely to have a first or a second birth than men in intact marriages. An additional analysis (not shown) indicated that when marital history was added to Model 2 before adjustments for occupational position and income, the remaining differences by educational level in the number of children and first-birth likelihood attenuated by more than two-thirds, whereas the respective attenuation in subsequent births was again small.

Table 4 shows the results from the sibling FE analysis. In the standard models (Models 0–2) of the different fertility outcomes run in the subsamples used for FE estimation, the estimates of education were mainly similar to those of the whole study population. The estimates of education on the number of children were similar in the FE model and in Model 2 and thus showed no evidence that characteristics shared by brothers would confound the estimates. In addition, the FE models on the likelihood of a first and second birth indicated that among brothers a higher level of education predicted higher chances of a first and, to a smaller extent, a second birth. Significant educational differences in third-birth likelihood were found neither in Model 2 nor in the FE model, but the point estimates were quite similar to those in Table 3.

## Discussion

### Main Interpretations

This study assessed whether the association between men’s education and their fertility can be explained by early socioeconomic and other characteristics or mediated by later socioeconomic characteristics. Another aim was to analyse how the socioeconomic characteristics of men across the life course associated with fertility among men. Fertility comprised the eventual number of children and chances of a first, second, and third birth. The study takes the previous literature further by showing that socioeconomic advantage across the life course associates with male fertility: among Finnish men born between 1940 and 1950, lower numbers of children were found among men from socioeconomically less-advantaged families and among those with a lower educational level, occupational position, and income level. In addition, concerning parity-related differences, education and several other characteristics in early life and adulthood related more strongly to the likelihood of a first than a second and, particularly, a third birth.

As for the number of children overall and for first births, the results are in line with previous studies from Nordic countries (e.g. Kravdal and Rindfuss 2008; Lappegård et al. 2011; Lappegård and Rønsen 2013), showing that a higher number of children and first birth rates are associated with educational and other socioeconomic advantages in adulthood in men. Similarity between the two fertility outcomes is expected: in a previous Finnish study, the educational gradient in the number of children was mostly due to different first-birth chances (Nisén et al. 2014a). Varying results outside Nordic countries (e.g. Miettinen et al. 2015) may reflect measurement issues but also true contextual influences. Our results regarding early socioeconomic characteristics differ from a recent Swedish study (Dahlberg 2015), which found no differences in first-birth likelihood by parental education among men, with the discrepancy potentially attributable to different study periods.

Second births were also predicted by higher educational levels and by other indicators of socioeconomic advantage, but to a smaller extent than first births. These findings coincide with earlier studies in similar institutional settings, which mainly find positive effects of the father’s education on higher-order births (Duvander and Andersson 2006; Duvander et al. 2010; Kravdal 2007; Kravdal and Rindfuss 2008; Lappegård and Rønsen 2013; Oláh 2003; Thomson et al. 2013). Similar to the present study, in some previous studies weaker effects of income (Lappegård and Rønsen 2013) and unemployment (Kravdal 2002) on higher-order births than on first births among men also appeared. In Denmark, unemployment depressed second birth rates overall less than first birth rates at older ages (Kreyenfeld and Andersson 2014). Besides economic factors, normative reasons, such as following the two-child norm, may be relatively important determinants of the transition to the second child (Bacci 2001; Goldstein et al. 2003), at least in the Finnish context (Miettinen 2015).

More favourable socioeconomic characteristics did not consistently associate with higher third-birth chances. This is similar to earlier Nordic studies showing that two-child fathers with weak labour market attachment (Andersson and Scott 2007; Kravdal 2002; Kreyenfeld and Andersson 2014) and low-earning couples were more likely to have a third child (Duvander and Andersson 2006; Duvander et al. 2010). After controlling for other socioeconomic characteristics (and marital history), a small positive effect of tertiary education on the chances of a third birth emerged in this study. Apart from economic reasons, two-child parents with high education may have relatively strong preferences for a larger number of children (Ruokolainen and Notkola 2002), which may show in their realized third-birth chances.

A novel finding was that the educational gradient in fertility in men was neither explained by early-life socioeconomic nor by other characteristics shared by brothers. These results reinforce the prevailing understanding, according to which mechanisms in later phases of the life course are more relevant in explaining the educational differences in men’s fertility. Indeed, men’s occupational position and income mediated approximately half of the association between education and the number of children. Differences in first births were strongly mediated by such factors. The corresponding mediation was more modest for second births, and only relatively small differences by education were found in the third-birth chances.

Education is a major determinant of long-term labour market position and income, whereas income and occupational position are proximate measures of economic standing, earning potential, and labour market attachment (Elo 2009; Lynch and Kaplan 2000). Given the current results, a plausible interpretation is that men’s economic potential creates differences between educational groups more strongly in the chance of having a first birth than in the chances of subsequent births. Men’s role as financial providers of the family may be more central for the process of entering parenthood than for subsequent childbearing. A similar interpretation may apply for results from another Nordic country, Norway (Lappegård and Rønsen 2013).

We view union formation and stability primarily as potential mediators of the association between education and fertility. Union experiences were measured by marital history; in the studied birth cohorts, marriage was still the normative context of childbearing (Finnäs 1993). Remarried men had the highest numbers of children (see also Van Bavel et al. 2012), but the largest fertility differences lay between the never married and other men. Marital history strongly mediated the chance of a first birth, but its inclusion as a model covariate had only a marginal effect for educational differences in subsequent births. Given the differences in other socioeconomic characteristics and marital history, those with tertiary education had 5% more children relative to those with basic education, but were not more likely to have a first child. This means that after accounting for such characteristics, the slightly higher fertility of the tertiary-educated group resulted from higher chances of second and third births.

Union formation selective on men’s potential as economic providers is thus likely to contribute to the educational differences in the number of children fathered by men through entry into parenthood. Along the lines of economic reasoning, men’s potential as economic providers is an important precondition for setting up an independent household (Oppenheimer 1988; Oppenheimer et al. 1997) and having children (Becker 1993). Therefore, women are likely to view men with actual or prospective higher incomes and better positions in the labour market as more attractive partners and potential fathers of their future children. Those educational differences in the second- and third-birth chances of men that were witnessed after accounting for their other observed socioeconomic characteristics, and marital history could be attributable to other factors. In the Finnish context, these could include varying strength of the two-child norm across educational groups, stronger preference-based selection into parenthood among more highly educated female partners (Kravdal 2001; Kreyenfeld 2002), and other such characteristics of female partners likely to vary between educational groups of men.^7^

The division of labour in families was less decisive in Finland than in many other Western countries in the 1960s and 1970s during the prime childbearing years of the studied men. Dual-earner families were increasingly common in Finland after the 1950s, and the wife’s income, often from full-time work, contributed an important share to the family income (Julkunen 1999). Still, a larger share of family income was earned by men in most dual-earner families (Aarnio and Eriksson 1987). Gender roles also remained asymmetrical in household and caring responsibilities; for instance, in the 1980s, less than 5% of Finnish men used their right to parental leave (Ellingsaeter and Leira 2006). Among Finnish women born between 1940 and 1950, educational level associated negatively with their eventual number of children (Nisén et al. 2014b). Besides gendered union patterns, fertility in educationally heterogamous unions may contribute to the gender difference. This point deserves more attention in future studies. Overall, the different associations among men and women may indicate gendered views of the breadwinner and caregiver roles in society, but also other issues such as a stronger effect of educational enrolment among women (Dribe and Stanfors 2009; Kravdal 2007).

An important issue regarding the interpretation of the parity-specific analysis is the selection of men into the risk group of a subsequent birth. Given that entering the first and, to a lesser extent, the second parity is selective on socioeconomic characteristics, the population at risk of a second or third birth is non-representative of the whole male population. For example, low-income men enter parenthood at a lower rate than high-income men, but those low-income men who do so may be a select group with additional characteristics that make them particularly attractive as partners and suitable as fathers. This may affect the results concerning subsequent parities, for example, by making some gradients less positive. A previous study used simultaneous modelling of first, second, and third birth rates to tackle this selection problem but concluded that this only marginally affected the estimates of education on men’s second and third births (Kravdal 2007). It is thus unlikely that such selection strongly drives the current results.

Another consideration in the interpretation of our results is related to children’s quality as opposed to their quantity (Becker 1993; Becker and Lewis 1973). Couples with more resources may restrict their subsequent fertility in order to guarantee sufficient resources for their earlier-born children. This could potentially contribute to some of the current parity-specific results. We cannot rule out this possibility but would consider the trade-off at least of less importance than direct considerations of affording to set up an independent household and have children. Importantly, we do not witness any strongly negative gradients by income or other socioeconomic characteristics in any fertility outcome.

Finally, men’s socioeconomic characteristics may also reflect characteristics such as health or problem-solving skills, which are correlated with education and may directly affect fertility, but which could not be measured here. The comparison of brothers was an attempt to more closely implement a causal research design: brothers partly share their social environment and genetic make-up. We found that neither observed nor unobserved characteristics shared by brothers explained the association between education and fertility in men, but brothers may still differ in relevant ways not captured here (Holmlund 2005; Kohler et al. 2011) that contribute to educational level and labour market success on the one hand and the chances of marrying and childbearing on the other. More research is welcomed on the relative importance of the different mechanisms behind educational differences in men’s fertility.

### Limitations

We consider the strengths of this study to include the large set of non-retrospectively measured socioeconomic variables from early life and the longitudinal measurement of men’s fertility based on administrative registers. Additionally, we view the sibling comparison as an innovative approach in research on men’s fertility. The limitations of this methodological approach should also be acknowledged, however. The method does not capture genetic or environmental endowments unshared by brothers, as, for example, siblings may be exposed differentially to the family environment due to effects of birth order and birth interval length (Kohler et al. 2011).

The study sample was limited in that men who died or emigrated between 1950 and 1970 or 1970 and 1985/1990/1995 were excluded. The men excluded from the sample prior to 1970 were more often born before 1945 or came from lower socioeconomic backgrounds, mother-only families, or Lapland (Elo et al. 2014). These differences were not large and thus unlikely to bias our main findings.

Children born before 1970 were registered only if co-residing with their fathers in the 1970 census. This may introduce a selective bias in our sample (Nelson, 2004). In our data, among women born in the early 1940s, living alone with children in 1970 was more common among those educated to lower levels. In the period 1966–1970, however, only 5% of children were reported to have been born out of wedlock in Finland (Finnäs 1993).

Our measurement of socioeconomic characteristics in adulthood is compromised by its non-time-varying nature. As a large share of fertility had occurred according to the measurement at the ages of 30–34, we face the risks of anticipatory analysis (Hoem and Kreyenfeld 2006). Although men’s later timing of and less intensive role in childbearing make children less likely to interfere with their educational careers compared to women (Woodward et al. 2006), the reverse causality remains plausible, particularly for income, due to incentives to support a family (Gupta et al. 2007; Lundberg and Rose 2002). In a sensitivity analysis using income measured at the ages of 40–44, the main results were changed only marginally, suggesting that the variable used reflects long-term income.

### Conclusion

Having higher education and other favourable socioeconomic characteristics across the life course associated positively with the eventual number of children among Finnish men born in 1940–1950 in a context where a dual-earner family model was increasingly dominant but men’s role as breadwinners still remained relatively strong. The findings further suggested that early-life socioeconomic or other characteristics shared by brothers do not explain the association of education with fertility in men. In turn, income and labour market position appear as substantial mediators of the association of education with the chance of a first birth. Educational differences regarding second births were smaller and the respective mediating role of other characteristics weaker. A small differentiation by education and other socioeconomic characteristics was present for third births overall. The findings indicate that men’s potential as economic providers appears to be more decisive for their entry into parenthood than for their subsequent childbearing and that this potential is a major determinant of educational differentials in the number of children among men. It appears that early-life socioeconomic circumstances are not insignificant even for men’s eventual fertility, but socioeconomic characteristics in adulthood are more important. To conclude, the entry into fatherhood selective on men’s potential as economic providers contributes strongly to the positive association between education and the number of children among men.

## Electronic supplementary material

Below is the link to the electronic supplementary material.

Supplementary material 1 (PDF 165 kb)

Supplementary material 2 (PDF 164 kb)

Supplementary material 3 (PDF 164 kb)

## Footnotes

1 Apart from varying control variables, these studies differ in the follow-up, which may affect the results, i.e. the effect of educational level may be more positive if men are followed up to a higher age.

2 Furthermore, studies motivated by evolutionary theory and measuring both socioeconomic characteristics and the number of children at late reproductive ages do not find education after accounting for income to predict higher fertility, whereas income, respectively, predicts higher fertility in men (Barthold et al. 2012; Fieder and Huber 2007; Goodman and Koupil 2010; Hopcroft 2015; Nettle and Pollet 2008; Weeden et al. 2006).

3 A sensitivity analysis conducted on a sample of men born in 1943–1947 indicated that the main interpretations were not sensitive to this measurement issue.

4 Siblings who died or moved out before the 1950 census or those who were born thereafter were not covered.

5 Attenuation in percentages is calculated as (OR_Model4_ − OR_Model2_)/(OR_Model2_ − 1) × 100.

6 The interpretation was the same when a dichotomized indicator of marital history was used (ever vs never married).

7 Overall, female partners’ characteristics may contribute to observed differences, but analysing them requires a different analytical approach, as typically those without partners are excluded (Begall 2013; Jalovaara and Miettinen 2013).
