# Supplementary material for: Education, Other Socioeconomic Characteristics Across the Life Course, and Fertility Among Finnish Men
Source: Eur J Popul. 2017 Jul 27;34(3):337–66. doi: 10.1007/s10680-017-9430-8 (PMC6096873; doi:10.1007/s10680-017-9430-8)
Supplement: Supplementary file 1 — Supplementary material 1 (PDF 165 kb) [file 10680_2017_9430_MOESM1_ESM.pdf]

Supplementary material 1

Odds ratios (OR) of the likelihood of a first birth among Finnish men, N =37,082

Model	0		1		2		3		4		5	
	OR	(SE)	OR	(SE)	OR	(SE)	OR	(SE)	OR	(SE)	OR	(SE)
Level of education												
Basic (ref.)	1		1		1		1		1		1	
Lower secondary	1.45*	(0.021)	1.44*	(0.021)	1.42*	(0.021)	1.35*	(0.021)	1.25*	(0.033)	1.07	(0.042)
Upper secondary	1.90*	(0.027)	1.87*	(0.027)	1.84*	(0.028)	1.34*	(0.030)	1.19*	(0.052)	1.00	(0.065)
Tertiary	2.28*	(0.027)	2.24*	(0.028)	2.22*	(0.031)	1.41*	(0.038)	1.13	(0.070)	0.90	(0.084)
Living area in childhood												
Helsinki region	0.99	(0.034)	0.91	(0.034)	0.90	(0.037)	0.90	(0.038)	0.85*	(0.061)	0.84*	(0.075)
Rest of Uusimaa	0.96	(0.040)	0.99	(0.040)	0.97	(0.040)	0.95	(0.041)	0.91	(0.067)	1.00	(0.087)
Western Finland (ref.)	1		1		1		1		1		1	
Eastern Finland	0.83	(0.020)	0.86*	(0.020)	0.91*	(0.021)	0.92*	(0.022)	0.93*	(0.034)	1.00	(0.041)
Northern Finland	0.86*	(0.046)	0.88*	(0.046)	0.95	(0.047)	1.00	(0.047)	1.05	(0.071)	1.27*	(0.087)
Family type in childhood												
Two parents and children (ref.)	1		1		1		1		1		1	
Mother and children	0.80*	(0.030)	0.83*	(0.031)	0.84*	(0.032)	0.86*	(0.032)	0.87*	(0.057)	1.01	(0.069)
Father and children	0.65*	(0.077)	0.69*	(0.077)	0.72*	(0.077)	0.75*	(0.077)	0.76	(0.145)	0.77	(0.185)
Sibship size												
0 (ref.)	1		1		1		1		1		1	
1-2	1.07	(0.025)	1.07	(0.026)	1.08	(0.027)	1.08	(0.027)	1.05	(0.043)	1.03	(0.052)
3-	0.92	(0.027)	1.01	(0.028)	1.09	(0.031)	1.10	(0.032)	1.07	(0.050)	1.10	(0.060)
Parental level of education												
Less than primary (ref.)	1				1		1		1		1	
Primary school	1.37*	(0.024)			1.20*	(0.025)	1.17*	(0.025)	1.13*	(0.041)	1.12*	(0.052)
More than primary	1.66*	(0.034)			1.10	(0.044)	1.12	(0.044)	1.10	(0.069)	1.06	(0.084)
Parental occupational position												
Worker (ref.)	1				1		1		1		1	
Professional/administrative	1.16*	(0.026)			0.86*	(0.032)	0.83*	(0.033)	0.84*	(0.051)	0.81*	(0.059)
Farmer, <10 hect.	0.85*	(0.022)			0.91*	(0.025)	0.90*	(0.025)	0.93	(0.041)	0.96	(0.050)
Farmer, ≥10 hect.	1.03	(0.035)			0.94	(0.038)	0.92	(0.039)	0.93	(0.061)	0.99	(0.071)
Self-employed/other/unknown	1.09	(0.032)			1.04	(0.033)	1.04	(0.034)	1.06	(0.056)	1.06	(0.068)
Parental home ownership												
Owner (ref.)	1				1		1		1		1	
Renter	1.15*	(0.019)			1.01	(0.023)	1.01	(0.024)	1.01	(0.037)	1.04	(0.045)
Other/unknown	0.96*	(0.039)			0.97	(0.039)	0.97	(0.040)	0.97	(0.063)	0.98	(0.074)
Crowding in childhood												
< 2 (ref.)	1				1		1		1		1	
2 < 3	0.92*	(0.022)			1.00	(0.024)	0.99	(0.024)	0.99	(0.039)	1.00	(0.046)
≥ 3	0.77*	(0.022)			0.92	(0.027)	0.94	(0.027)	0.95	(0.044)	0.94	(0.051)
Standard of living in childhood												
Poor (ref.)	1				1		1		1		1	
Modest	1.27*	(0.021)			1.19*	(0.024)	1.09*	(0.024)	1.05	(0.039)	0.98	(0.047)
Good	1.49*	(0.024)			1.16*	(0.032)	1.13*	(0.033)	1.10	(0.054)	0.99	(0.064)
Occupational position												
Manual worker (ref.)	1						1		1		1	
Lower white collar	1.82*	(0.021)					1.62*	(0.023)	1.51*	(0.045)	1.23*	(0.060)
Upper white collar	2.25*	(0.029)					1.85*	(0.038)	1.56*	(0.068)	1.27*	(0.086)
Farmer/self-employed	1.08	(0.033)					1.08	(0.033)	1.48*	(0.050)	1.46*	(0.062)
Other/unknown	0.34*	(0.027)					0.33*	(0.028)	0.59*	(0.046)	0.76*	(0.058)
Income												
1st quintile	0.30*	(0.040)							0.37*	(0.043)	0.49*	(0.054)
2nd quintile	0.82*	(0.045)							0.91	(0.047)	0.91	(0.059)
3rd quintile (ref.)	1								1		1	
4th quintile	1.28*	(0.046)							1.24*	(0.047)	1.06	(0.057)
5th quintile	2.01*	(0.052)							1.72*	(0.053)	1.38*	(0.062)
Marital history												
Never-married	0.03*	(0.026)									0.04*	(0.040)
Intact married (ref.)	1										1	
Divorced/widowed	0.74*	(0.028)									0.83*	(0.049)
Remarried	1.17*	(0.042)									1.24*	(0.072)

Model 0: explanatory variable + year of birth. Calculated separately for each explanatory variable

Model 1: level of education + control variables

Model 2: Model 1 + socioeconomic characteristics in early life

Model 3: Model 2 + occupational position

Model 4: Model 3 + income

Model 5: Model 4 + marital history

Method: logistic regression analysis. In all models year of birth is included as a continuous variable, but the coefficient is not shown

SE standard error

An asterisk indicates when the 95% confidence interval does not include 1

Education, Other Socioeconomic Characteristics Across the Life Course and Fertility Among Finnish Men

European Journal of Population

Jessica Nisén[1,2], Pekka Martikainen[1,2,3,4], Mikko Myrskylä[1,2,5], Karri Silventoinen[1,6]

[1]Population Research Unit, Department of Social Research, University of Helsinki, P.O. Box 18 (Unioninkatu 35), 00014 Helsinki, Finland

[2]Max Planck Institute for Demographic Research, Konrad-Zuse-Straße 1, 18057 Rostock, Germany

[3]Centre for Health Equity Studies (CHESS), Stockholm University, Stockholm, Sweden

[4]Karolinska Institutet, Stockholm, Sweden

[5]Department of Social Policy, London School of Economics, London, UK

[6]School of Medicine, Osaka University, Suita, Japan

jessica.nisen@helsinki.fi; nisen@demogr.mpg.de
