# Supplementary material for: Education, Other Socioeconomic Characteristics Across the Life Course, and Fertility Among Finnish Men
Source: Eur J Popul. 2017 Jul 27;34(3):337–66. doi: 10.1007/s10680-017-9430-8 (PMC6096873; doi:10.1007/s10680-017-9430-8)
Supplement: Supplementary file 3 — Supplementary material 3 (PDF 164 kb) [file 10680_2017_9430_MOESM3_ESM.pdf]

Supplementary material 3

Odds ratios (OR) of the likelihood of a third birth among Finnish fathers of at least two children, *n* =23,152

Model	0		1		2		3		4		5	
	OR	(SE)	OR	(SE)	OR	(SE)	OR	(SE)	OR	(SE)	OR	(SE)
Level of education												
Basic (ref.)	1		1		1		1		1		1	
Lower secondary	0.88*	(0.034)	0.89*	(0.034)	0.89*	(0.034)	0.92*	(0.034)	0.93*	(0.034)	0.93*	(0.035)
Upper secondary	0.80*	(0.041)	0.82*	(0.042)	0.83*	(0.043)	0.91*	(0.049)	0.92	(0.049)	0.92	(0.050)
Tertiary	1.03	(0.040)	1.07	(0.040)	1.07	(0.044)	1.14*	(0.058)	1.17*	(0.059)	1.20*	(0.060)
Living area in childhood												
Helsinki region	1.00	(0.051)	1.02	(0.051)	1.05	(0.055)	1.05	(0.055)	1.06	(0.055)	1.01	(0.056)
Rest of Uusimaa	0.84	(0.062)	0.84*	(0.063)	0.84*	(0.063)	0.84*	(0.063)	0.85*	(0.063)	0.84*	(0.064)
Western Finland (ref.)	1		1		1		1		1		1	
Eastern Finland	1.09	(0.030)	1.06*	(0.030)	1.06	(0.032)	1.06	(0.032)	1.06	(0.032)	1.06	(0.032)
Northern Finland	1.18	(0.067)	1.15*	(0.068)	1.14	(0.069)	1.14	(0.069)	1.13	(0.069)	1.14*	(0.070)
Family type in childhood												
Two parents and children (ref.)	1		1		1		1		1		1	
Mother and children	0.92	(0.056)	0.98	(0.057)	0.96	(0.058)	0.98	(0.058)	0.98	(0.058)	0.97	(0.058)
Father and children	0.81	(0.167)	0.85	(0.167)	0.83	(0.168)	0.83	(0.169)	0.82	(0.168)	0.77	(0.172)
Sibship size												
0 (ref.)	1		1		1		1		1		1	
1-2	1.14*	(0.041)	1.13*	(0.041)	1.13*	(0.042)	1.13*	(0.043)	1.14*	(0.043)	1.14*	(0.043)
3-	1.33*	(0.044)	1.30*	(0.045)	1.24*	(0.049)	1.25*	(0.049)	1.26*	(0.049)	1.27*	(0.049)
Parental level of education												
Less than primary (ref.)	1				1		1		1		1	
Primary school	0.87*	(0.043)			0.93	(0.045)	0.93	(0.045)	0.94	(0.045)	0.94	(0.045)
More than primary	0.98	(0.054)			1.15*	(0.068)	1.14	(0.068)	1.15	(0.068)	1.13	(0.069)
Parental occupational position												
Worker (ref.)	1				1		1		1		1	
Professional/administrative	1.00	(0.039)			0.94	(0.048)	0.93	(0.048)	0.93	(0.048)	0.92	(0.049)
Farmer, <10 hect.	1.15*	(0.035)			1.10*	(0.040)	1.06	(0.040)	1.05	(0.040)	1.08	(0.041)
Farmer, ≥10 hect.	1.23*	(0.051)			1.23*	(0.055)	1.11	(0.056)	1.11	(0.057)	1.16*	(0.057)
Self-employed/other/unknown	1.11*	(0.048)			1.11*	(0.051)	1.08	(0.051)	1.08	(0.051)	1.08	(0.052)
Parental home ownership												
Owner (ref.)	1				1		1		1		1	
Renter	0.90*	(0.029)			0.99	(0.036)	1.00	(0.036)	1.00	(0.036)	0.99	(0.036)
Other/unknown	0.96	(0.061)			0.98	(0.061)	0.99	(0.062)	0.99	(0.062)	0.98	(0.063)
Crowding in childhood												
< 2 (ref.)	1				1		1		1		1	
2 < 3	0.99	(0.034)			0.98	(0.036)	0.99	(0.037)	0.99	(0.037)	0.99	(0.037)
≥ 3	1.12*	(0.032)			1.07	(0.040)	1.09*	(0.041)	1.08*	(0.041)	1.08*	(0.041)
Standard of living in childhood												
Poor (ref.)	1				1		1		1		1	
Modest	0.92*	(0.033)			1.02	(0.037)	1.02	(0.037)	1.02	(0.037)	1.02	(0.037)
Good	0.87*	(0.037)			0.96	(0.047)	0.94	(0.047)	0.95	(0.048)	0.94	(0.048)
Occupational position												
Manual worker (ref.)	1						1		1		1	
Lower white collar	0.89*	(0.036)					0.94	(0.041)	0.94	(0.041)	0.93	(0.041)
Upper white collar	1.08*	(0.037)					1.02	(0.054)	1.03	(0.055)	1.01	(0.056)
Farmer/self-employed	1.55*	(0.044)					1.54*	(0.046)	1.45*	(0.047)	1.48*	(0.048)
Other/unknown	1.36*	(0.060)					1.34*	(0.061)	1.19*	(0.064)	1.13*	(0.064)
Income												
1st quintile	1.40*	(0.044)							1.27*	(0.047)	1.28*	(0.048)
2nd quintile	0.97	(0.042)							0.97	(0.043)	0.98	(0.043)
3rd quintile (ref.)	1								1		1	
4th quintile	0.92*	(0.041)							0.94	(0.042)	0.94	(0.042)
5th quintile	0.95	(0.041)							0.94	(0.043)	0.93	(0.043)
Marital history												
Never-married	0.91	(0.063)									0.89	(0.064)
Intact married (ref.)	1										1	
Divorced/widowed	1.03	(0.034)									1.04	(0.035)
Remarried	2.04*	(0.040)									2.16*	(0.041)

Model 0: explanatory variable + year of birth. Calculated separately for each explanatory variable

Model 1: level of education + control variables

Model 2: Model 1 + socioeconomic characteristics in early life

Model 3: Model 2 + occupational position

Model 4: Model 3 + income

Model 5: Model 4 + marital history

Method: logistic regression analysis. In all models year of birth is included as a continuous variable, but the coefficient is not shown

SE standard error

An asterisk indicates when the 95% confidence interval does not include 1

Education, Other Socioeconomic Characteristics Across the Life Course and Fertility Among Finnish Men

European Journal of Population

Jessica Nisén[1,2], Pekka Martikainen[1,2,3,4], Mikko Myrskylä[1,2,5], Karri Silventoinen[1,6]

[1]Population Research Unit, Department of Social Research, University of Helsinki, P.O. Box 18 (Unioninkatu 35), 00014 Helsinki, Finland

[2]Max Planck Institute for Demographic Research, Konrad-Zuse-Straße 1, 18057 Rostock, Germany

[3]Centre for Health Equity Studies (CHESS), Stockholm University, Stockholm, Sweden

[4]Karolinska Institutet, Stockholm, Sweden

[5]Department of Social Policy, London School of Economics, London, UK

[6]School of Medicine, Osaka University, Suita, Japan

jessica.nisen@helsinki.fi; nisen@demogr.mpg.de
